# A Systematic Review of Abuse or Overprescription of Bupropion in American Prisons and a Synthesis of Case Reports on Bupropion Abuse in American Prison and Non-prison Systems

**DOI:** 10.7759/cureus.36189

**Published:** 2023-03-15

**Authors:** Salisu Aikoye, Tajudeen O Basiru, Idorenyin Nwoye, Iyanujesu Adereti, Sarah Asuquo, Adaobi Ezeokoli, Jessie Hardy, Osaretin Umudi

**Affiliations:** 1 Psychiatry, Charles R. Drew University of Medicine and Science, Los Angeles, USA; 2 Psychiatry, Community Health of South Florida, Miami, USA; 3 Pulmonary Medicine, IQVIA Global Solutions, Los Angeles, USA; 4 Psychiatry, CIT Center of Excellence Clinical Research, Riverside, USA; 5 Psychiatry, University of North Carolina, Chapel Hill, USA; 6 Psychiatry, Gateway Behavioral Health Services, Savanna, USA; 7 Psychiatry, Elmhurst Hospital Center, Queens, USA; 8 Psychiatry, Meharry Medical College, Nashville, USA

**Keywords:** case report synthesis, adult, systematic review, american prisons, abuse

## Abstract

Illicit drug use is a huge problem in the US prison system. The objectives of this study are (1) to systematically investigate the prevalence of bupropion abuse in American prisons along with associated problems, and (2) to synthesize available case reports on this topic in both prison and non-prison settings. Using the Preferred Reporting Items for Systematic Reviews and Meta-analyses, we searched five databases (PubMed, Embase, Scopus, CINAHL, and PsycINFO) and used Covidence software for screening and reviewing identified articles. The final search date was February 21, 2023. Newcastle-Ottawa Scale and ROBINS-I tool were used for risk of bias assessment. We included original studies of populations aged 18 years and above in American prisons. We found 77 unique articles, none of which met our eligibility criteria. A synthesis of 22 case reports that we found showed bupropion abuse to be more common in young males, and intranasal administration was the most common method of abuse. More frequent desired and adverse effects were “cocaine-like highs” and seizures, respectively. Although several cases of bupropion abuse have been reported in US prisons, no study has been done to understand its prevalence and associated effects. The absence of original studies on bupropion abuse in US prisons and the observed patterns in this case report synthesis further highlight the need for a study to investigate the prevalence of bupropion abuse in US prisons. The limitations of this study include that it is an empty systematic review and the absence of all pertinent data in many of the included case reports. The authors did not receive any funding for this work. This systematic review was registered in PROSPERO with registration number CRD42021227561.

## Introduction and background

About 1% of the US adult population is incarcerated [1], while one in every 31 US adults was under some form of incarceration or supervision such as parole at the end of 2013 [2]. According to the Criminal Justice fact sheet published by the National Institute on Drug Abuse (NIDA), in June 2020, about 85% of the US prison population had an active substance use disorder or were imprisoned for a drug or drug use crime [3]. Drug abuse in the US is currently on an upward trend. The National Survey on Drug Use and Health (NSDUH) reported that illicit drug use among persons 12 years or older increased from 17.8% (or 47.7 million people) in 2015 to 20.8% (or 57.2 million people) in 2019 [4]. The associated physical and psychosocial health consequences of drug abuse include premature death, cardiovascular and liver pathology [5], depression, suicidal behaviors, engagement in violent crimes [6], and sleep abnormalities [7]. In emerging adulthood, drug abuse has been linked to difficulty with interpersonal relationships, problems sustaining employment, and lower expectations for success in life [8,9]. Thus, combating this rising trend in drug abuse is critical to sustaining optimal population health, as well as individual and national economic advancement.

American prisons are also affected by the high prevalence of drug abuse. A 2017 Bureau of Justice Statistics report put the proportion of sentenced prison inmates who met the Diagnostic and Statistical Manual of Mental Disorders (DSM) intravenous (IV) drug dependence/drug abuse criteria between 2007 and 2009 at 63% and those of state prisoners at 58% [10]. However, only one in 20 adults aged 18 years and older in the general population met the DSM IV drug abuse/dependence criteria. A 2006 systematic review of 13 studies [11] consisting mainly of US prisoners (6,635 US prisoners, 88%) found a varying prevalence of substance abuse ranging between 10% in a 1988 study and 60% among females in a 2000 study. Although the result of the study indicated a considerably high variability in the prevalence of substance abuse in prisons, the important finding from the study was that drug abuse/dependence prevalence was significantly higher among prison populations than the general population [11]. Bupropion abuse/overprescription is a rising problem in US prisons.

Bupropion (marketed as Wellbutrin or Zyban) is an aminoketone that belongs to the class of atypical antidepressants structurally similar to diethylpropion, an appetite suppressant. The mechanism of the antidepressant effect of bupropion is not fully understood, but it is a norepinephrine, dopamine reuptake inhibitor (NDRI) antidepressant [12]. Bupropion is also a non-competitive nicotine receptor antagonist, which at high concentrations inhibits the firing of noradrenergic neurons in the locus coeruleus. It is a prescription medication used to treat major depression and seasonal affective disorder. Bupropion is the only NDRI that is approved by the FDA to treat depression and is as effective as other antidepressants, yet without the common side effects associated with most antidepressants [13]. Other uses include smoking cessation as well as off-label uses such as treatment of sexual dysfunction caused by antidepressants, obesity, attention-deficit hyperactivity disorder (ADHD), and bipolar disorder-associated depression [14]. Bupropion is particularly important in the prison population where about 48% have symptoms of mental illness, with major depressive disorders being the most prevalent [15]. About 18% of all inmates in American prisons have depression which accounts for 38% of all mental illnesses in this population [15].

There are limited studies on bupropion abuse although there is an increase in the number of case reports citing its abuse in the general population [16]. There are also reports of bupropion abuse in the American prison system where it is sometimes referred to as “poor man’s cocaine” as it is said to give a “high” that is similar to that experienced on using cocaine or amphetamine [17,18]. It is most commonly ingested either by crushing or insufflated nasally [19]. There are scant studies on the prevalence of bupropion abuse in American prisons or in the general population [16,20]. In this study, we attempted to determine the prevalence and associated effects of abuse and overprescription of bupropion in US prisons by conducting a systematic review of the literature. We also conducted a synthesis of all available case reports on bupropion abuse in prison and non-prison settings.

## Review

Methodology

The authors adhered to the Preferred Reporting Items for Systematic Reviews and Meta-analyses (PRISMA) guidelines for conducting and reporting this systematic review [21]. A study protocol was developed and registered on PROSPERO with registration number CRD42021227561 (see Appendices).

Search Strategy

On January 6, 2021, the authors searched the following five literature databases: PubMed, Embase, Scopus, CINAHL, and PsycINFO. Keywords and medical subject headings (MeSH) associated with substance abuse, prisons, and bupropion or Wellbutrin were used in the search (see Appendices). We also searched the same databases and Google Scholar for case reports and extracted case report bibliographies from a systematic review by Naglich et al. [22]. The search was conducted again on February 21, 2023, and found no new studies meeting our inclusion criteria.

Inclusion and Exclusion Criteria

Inclusion criteria for articles to be reviewed included study population aged 18 years and above who were in the American prison system, articles published in the English language, and articles reporting at least one of the following outcomes: prevalence of bupropion overprescription, the prevalence of bupropion abuse, sources of abused or misused bupropion, and medical, mental health, societal, and economic effects of bupropion abuse. Studies were excluded if they were not original studies, if they were literature reviews, if the population studied was not of people in the American prison system, or if they did not report at least one of the outcomes mentioned above.

Data Extraction, Risk of Bias Assessment, and Data Synthesis

We used Covidence, a web-based software for screening and full-text review. All authors were involved in the title/abstract screening and full-text review, and each article was independently reviewed by any two of the authors; conflicts were resolved by a third author. Data for extraction were population attributes (age, gender, race, ethnicity, educational level), sample size, setting type, study design, outcomes of interest, methods of outcome measurement, the prevalence of bupropion abuse/overprescription through legal prescriptions, prevalence/proportion of bupropion abuse gotten through non-prescriptions, and effects of bupropion abuse/overprescription. We planned to conduct a risk of bias assessment of selected articles using the Newcastle-Ottawa Scale (NOS) and ROBINS-I tool [23]. Data synthesis was to be done by qualitative analysis of the following extracted data: source of bupropion (prescription or non-prescription), indication for use (depression or smoking cessation), comorbidity status (co-existing medical condition, history of substance abuse), co-existing substance use disorder, criminal history, and effects of bupropion abuse/overprescription.

Results

Our search strategy yielded 77 unique citations. At the end of the title/abstract screening, only one article made it to the full-text review, but it was finally excluded because it was a literature review. None of the articles met the eligibility criteria for this review, highlighting the importance of this study. However, we identified 22 cases in 18 case reports which we synthesized, analyzed, and discuss below.

Synthesis of Case Reports

Table 1 describes the features and patterns seen in reported cases of bupropion abuse as discussed below.

**Table 1:** Synthesis of case reports. *: More than one feature seen in many patients. ^a^: Each feature had the same frequencies. ADHD = attention-deficit hyperactivity disorder; PTSD = post-traumatic stress disorder; ARDS = acute respiratory distress syndrome

Characteristics		N (%)
N = 22		
Age (years)		
	Mean (SD)	32.1 (16.56)
	Median age (IQR)	29.5 (27)
	Mode	16
	Range	13–79
	Children (less than 18 years)	7 (31.8)
	Adult (18 years and older)	15 (68.2)
Gender		
	Male	16 (72.7%)
	Female	6 (27.3%)
Route of administration		
	Intranasal	11 (50%)
	Oral	7 (31.8%)
	Intravenous	2 (0.1%)
	Unknown	2 (0.1%)
Setting		
	Home	9 (40.9%)
	Prison	3 (13.6%)
	School	1 (<0.1%)
	Others	3 (13.6%)
Comorbidities*		
	Substance use (cocaine, alcohol, amphetamine, opioid, tobacco, etc.)	14 (63.6%)
	Mood disorder (depression, bipolar disorder, schizoaffective disorder)	12 (54.5%)
	Others (ADHD, PTSD, panic attacks, anti-social personality disorder)	10 (45.5%)
Desired effect		
	Some high (cocaine)	4 (18.2%)
	Not mentioned	3 (13.6%)
	Some high (amphetamine), get high, depression treatment, brief buzz	2 (0.1%)
	Euphoric/stimulant like	1 (<0.1%)
	Others: altered reality, “get messed up,” bipolar disorder treatment, possible recreational use.	1 (<0.1%)^a^
Adverse effects*		
	Seizure	8 (36.4%)
	Hallucination, reduced need for sleep/insomnia, increased energy, intranasal bleed/nasal pain, paranoia, withdrawal, tachycardia, irritability, hypertension, not mentioned	2 (0.1%)^a^
	Grandiose thoughts, hypersexuality, pressured speech, malaise, numbness, labile mood, spells of anger, sleep disturbance, psychomotor agitation, restless leg, muscle pain, increased appetite, relapse of depression (when used with cocaine), panic attacks, vegetative dysregulation, overstimulation (cocaine), fidgety, impulsivity, aggressiveness, altered consciousness, fatigue, respiratory distress, confusion, ARDS, death, no effect	1 (<0.1%)^a^

Demographics

The ages of patients in the case reports ranged from 13 to 79 years, with 16 years appearing most frequently (three times, 13.2%). The mean (SD) and median (IQR) ages were 32.1 (16.56) years and 29.5 (27) years, respectively. Children aged less than 18 years appeared in 31.8% of the cases while 16 (72.7%) cases were less than 40 years old. Sixteen (72.7%) patients were males [13-25] while six (27.3%) were females [9,13-17]. One of the patients was Caucasian and homeless [24] while other demographic parameters of most cases were not mentioned in the case reports.

Pattern of Use

The most common route for bupropion use was nasal insufflation (seen in 11 cases ) [24-34], followed by the oral route (seen in seven cases) [35-38]. IV administration was observed in two cases [20,39], while two other cases did not report the route of administration [28,40]. Reported single doses of bupropion ranged from 900 to 6,750 mg orally or by nasal insufflation. IV administration was multiple times daily, with a daily dose of 1,200 to 2,400 mg daily. Some case reports mentioned patient location at the time of abuse or overdose: three cases occurred in prisons [27,28], eight cases were at home [29,30,33,35,40], two cases were in school [32,35], one case was homeless [24], while one case was in a transition home [25]. In two other cases, the patients said they learned about abusing bupropion when they were jailed [25,26]. There was one case where the patient did not misuse his prescribed bupropion but reported feeling a high similar to taking cocaine [37]. Another patient increased his prescribed dose on his own from two times to three times daily following worsening depressive symptoms [37].

Adverse/Desired Effects

Among the 22 case reports reviewed, more than one adverse effect was reported for each case while only one desired effect was reported for most cases. The most common (four cases) desired effect of abusing bupropion was a cocaine-like high (some high similar to cocaine) [13,14,24,28] while amphetamine-like high, (some high similar to amphetamine), “get high,” depression treatment, possible recreational use, and “brief buzz” were other common reasons why patients abused bupropion (two to three cases reported each of these) [15-17,20-22,24,25-27]. One patient reported a euphoric/stimulant-like effect [20]. Other less common desired effects (appeared in one case each) included altered reality, “getting messed up,” and bipolar disorder treatment [24,26].

A seizure was the most commonly reported adverse effect (eight cases reported some form of seizure) [24,25,29,31,33,35]. Four of these were reported as either generalized tonic-clonic seizure, generalized seizure, or tonic-clonic seizure [25,29,33,35], while the other four cases did not specify the type of seizures the patients experienced. Hallucination, reduced need for sleep/insomnia, increased energy, intranasal bleed/nasal pain, paranoia, withdrawal, tachycardia, irritability, and hypertension were reported in two cases each, while no adverse effects were reported in two cases [9,15-17,19,21,22,24,25,28]. Other less commonly experienced adverse effects included grandiose thought, hypersexuality, pressured speech, malaise, numbness, labile mood, spells of anger, sleep disturbance, psychomotor agitation, restless leg, muscle pain, increased appetite, relapse of depression (when used with cocaine), panic attacks, vegetative dysregulation, overstimulation, fidgety, impulsivity, aggressiveness, altered consciousness, fatigue, respiratory distress, confusion, acute respiratory distress syndrome, death, and no effect, each of which was reported only in one case [16,19,21-29].

Comorbidities

The most common psychiatric condition seen in our case report synthesis was substance use disorder, which included cocaine (six cases) [24,26,27,30,37], opioids (three cases) [20,26,34], amphetamines/methamphetamine (three cases) [27,36,37], and alcohol (three cases) [26,31,37]. Other psychiatric comorbidities and clinical signs comprised bipolar disorder (three cases) [20,25,37] and depression (seven cases) [20,26-28,30,36,39]. Conduct disorder [38], ADHD [32], schizoaffective disorder [24,28], social anxiety disorder [35], panic disorder [30], antisocial personality disorder [28], pathological gambling [37], post-traumatic stress disorder [25], and unassessed auditory hallucinations [35] were reported in one or two cases.

Case Reports in Prison Settings

Several cases of bupropion abuse have been reported in the US, with only a few being reported in prison settings. Naglich et al. reported about 18 cases of bupropion abuse spanning the years 2002 and 2015 mostly from non-peer-reviewed channels [22]. Only one case among the 18 cases summarized by Naglich et al. was reported in a prison clinic setting while the author noted that no published cases of bupropion abuse had been noted in prison settings [22]. However, two case reports described two cases where the patients started bupropion abuse when they were incarcerated, while Hilliard et al. also reported two additional cases of suspected bupropion abuse among incarcerated people [15,17]. Despite the reported concerns regarding bupropion abuse in prison settings, there is a dearth of peer-reviewed articles or scientific evidence to back this claim. Naglich et al. suggest the sensitive nature of privacy and confidentiality with regard to healthcare in prisons as being responsible for the difficulties in researching and publishing drug abuse in prison settings. Moreover, health research involving incarcerated people has a controversial history because of the vulnerability of this population to abuse. It is, therefore, not surprising that very few research and case reports among prisoners have been published as healthcare institutions have strict protocols that likely disincentivize reporting and researching among the population. However, the case from a prison clinic setting described by Reeves and Ladner in 2013 provides a glimpse into what bupropion abuse might look like in prisons [27].

According to Reeves and Ladner, the 49-year-old male inmate of a southeastern US prison reported that his hallucinations began when he started abusing bupropion prescribed for his depression, which he claimed previously defied conventional selective serotonin reuptake inhibitor (SSRI) treatment. He went on to disclose that he, like many other inmates, preferred bupropion to SSRIs because of the sexual side effects of SSRIs. Other important findings were his claim that bupropion abuse was common among prison inmates, his knowledge of five other inmates abusing bupropion, internal investigations in the prison that found three other inmates abusing bupropion with one of them possessing 33 pills of bupropion at the time, and his assertion that bupropion abuse was common in many other correctional facilities that he had knowledge of [41]. Table 2 summarizes the reported cases and observed features of bupropion abuse.

**Table 2:** Analysis of case reports.

Authors (years)	Age	Sex	Dose/form	Route of use	Setting	Desired effect	Adverse effects of abuse
Rahman et al., 2013 [35]	16	M	5 tablets - dose not known	Oral	At home	“Get high”	Generalized tonic-clonic seizure, frank hallucinations, altered mental status
Rahman et al., 2013 [35]	17	M	Tablets. Dose unknown	Oral	At home (possibly) - not stated	Experience alternate reality	Seizure
Rahman et al., 2013 [35]	16	F	A 10-day dose of tablets	Oral	School	“Get messed up”	Seizure
Rostas and Wolf, 2015 [36]	79	M	900 mg/day	Oral		Initially to improve the symptoms of depression. Later “get a less intense version of high gotten from amphetamine”	Euphoric mood, decreased need for sleep, increased energy, increased activity, and hypersexuality. Hypertension, pressured speech, euphoric affect, grandiose thought content
Kim and Steinhart, 2010 [25]	38	M	1,500 mg/use tablets (crushed)	Intranasal	Transition home	Get a euphoria like that attained from cocaine	Repeated generalized seizures
Yoon and Westermeyer, 2013 [26]	44	M	2,100 mg/day	Intranasal		Get high	Intranasal bleeding, malaise, numbness, paranoia
Vento et al., 2013 [37]	28	M	150 mg/day tablets	Oral		Depression treatment	Thought acceleration, restlessness, “high similar to that of cocaine”
Vento et al., 2013 [37]	56	M	150 mg/tid tablets	Oral		Bipolar treatment	Overstimulation, the same effect as cocaine
Hill et al., 2007 [24]	50	M	Sustained-release tablets. 150 mg/bid but abused dosage not quantified	Nasal insufflation	Homeless; on the streets	Chemical euphoria “cocaine high”	Seizure
Reeves and Ladner, 2013 [27]	49	M	100 mg/bid tablets. Snorts 4–10 or 12 tablets a day	Nasal insufflation	Correctional facility - prison	Depression	Auditory hallucination, insomnia, spells of anger
Oppek et al., 2014 [39]	29	F	2,400 mg/day	Intravenous (1.5 months)	Not stated	“Get high similar to cocaine of poor quality”	Paranoia, labile mood, sleep disturbance increased energy, withdrawal symptoms, psychomotor agitation, restless leg, muscle pain, ravenous appetite
Hilliard et al., 2013 [28]	15	M	150 mg/day prescribed. Abused dose not mentioned	Route not ascertained	Prison	Possible recreational use	None mentioned
Hilliard et al., 2013 [28]	43	M	200 mg/bid prescribed. Abuse dose not mentioned	Intranasal	Prison cell	Possible recreational use	None mentioned
Anderson, 2019 [29]	33	M	Not mentioned	Nasal insufflation	Home	Not mentioned	Tonic-clonic seizure
Baribeau and Araki, 2013 [20]	29	F	1,200 mg daily (4 × 300 mg)	Intravenous	Home (possibly) - not clear	Comfort from injection drug use. Euphoric and stimulant-like effect	Irritability, lability, low mood during periods of abstinence
Langguth et al., 2009 [30]	23	F	Eight tablets of 150–1,200 mg	Nasal insufflation	Home	Treatment of depressive symptoms but abused it to experience the stimulating effect of cocaine	Local pain in the nose but when combined with cocaine experienced relapse of depressive symptoms, increased frequency of panic attacks, vegetative dysregulations with tachycardia
McCormick 2002 [38]	13	F	Four tablets of 600 mg	Orally		Amphetamine high	Developed no adverse effect
Welsh and Doyon, 2002 [31]	16	M	Six tablets of 150 mg (sustained-released) – 900 mg	Nasal insufflation		Brief “buzz”	Seizures
Khurshid and Decker, 2004 [32]	15	F	150 mg sustained-released tablets but abused dosage not quantified	Nasal insufflation	Steals from home to snort at school with friends	Prescribed for ADHD but gets a brief “buzz” when abused	Increased irritability, fidgeting, impulsivity, and aggressive behavior at home
Rigatti, 2019 [33]	33	M	Maximum potential dose up to 6.75 g to 6,750 mg	Possible nasal insufflation	Home	Possibly to get high, per the psychiatrist	Generalized seizure (two episodes), altered consciousness, non-extinguishing clonus of lower extremities, mild hypertension, tachycardia
Al-Saiegh et al., 2020 [34]	30	M	Not mentioned	Intranasal	Not mentioned	Not mentioned	Extreme fatigue, respiratory distress, confusion. ARDS confirmed with CT scan
Mercerolle et al., 2008 [40]	35	M	Empty box of Zyban found near the body, (30 sustained-released tablets of 150 mg each) – 4,500 mg	Unknown	Home	Not mentioned	Death

The 44-year-old described by Yoon and Westermeyer in a prison setting was said to have learned to abuse bupropion after spending a night at a local jail for driving under influence [26]. He subsequently continued to abuse bupropion for the next 10 weeks, which he said produced approximately one-fifth of the “high” produced by cocaine, before reporting to an outpatient psychiatric clinic. One of the reasons he continued with bupropion was because he needed to have negative urine drug tests to be able to continue his opioid dependency treatment and so bupropion, being less potent than cocaine, afforded him that. The 38-year-old man described by Kim and Steinhart experienced three episodes of seizures and visited the emergency department (ED) twice. At his first visit, his nose examination was omitted but was found to have a powdery substance in his fingers and nostrils during the second ED visit. He reported that he learned to abuse bupropion when he was incarcerated and that bupropion abuse “was commonplace” in the prison [25]. The two cases reported by Hilliard et al. were suspected cases as the patients denied bupropion abuse [28]. One of the patients, a 15-year-old with a history of cannabis use who was in jail for aggravated assault, complained about depressive symptoms and was not responsive to Prozac and Celexa despite being treatment-naive. He was subsequently started on bupropion only to be found hiding a bupropion pill in his shoes two weeks later. He denied being aware of the presence of the drug and his bupropion treatment was discontinued for suspected diversion. The other patient was a 43-year-old serving terms for sexual assault who complained of depression and claimed that many antidepressants did not work for him except a drug “he cannot recall the name, but it started with a ‘W’.” One month after bupropion was prescribed for the patient, he was found snorting a white powder suspected to be bupropion and his urine screening was positive for amphetamine.

Discussion

In this systematic review of empirical evidence regarding reports of increasing cases of bupropion abuse in American prisons, no articles met our inclusion criteria. While limited studies exist on the abuse of bupropion in prisons [22], a review of the available studies showed that bupropion is a drug with significant abuse potential and there is a rising number of reported cases of abuse [17]. Although not a controlled substance by the US FDA, labeling of commercial bupropion packages cautions about the abuse potential of the drug [22]. Different psychomotor tests that are used to assess investigational drugs for abuse potentials in animal studies have been performed on bupropion and some support the abuse potential of bupropion [42-45].

Although the proportion of the US population that is in prison is estimated to be 0.7% [1], our case report synthesis showed that 22.7% (five cases) of all published case reports of bupropion abuse occurred in or were related to prison settings. However, the prevalence of bupropion abuse among the incarcerated population is unknown at the moment [46]. For data on the general population in the US, retrospective data analysis of cases reported to the Toxicology Investigators Consortium (ToxIC) between 2015 and 2017 revealed a 36% increase in single-agent bupropion cases per 1,000, 9% of which were due to abuse/misuse [47]. Another 14-year review of the cases reported to the US National Poison Data System indicated an increase in the prevalence of bupropion abuse by 75% between 2000 and 2012 [16]. Adolescents and young adults accounted for the most reported cases (67.4%). Our case report synthesis showed similar results as over 70% of the cases were less than 40 years old.

From the findings of this case report synthesis, there is an apparent relationship between bupropion misuse and substance use, particularly cocaine and amphetamine. In two of our case reports, the patients stated that the high from bupropion was similar to cocaine or amphetamine use [37,39], although less potent, and some patients were current or recovering cocaine addicts [24,26,27,30,37]. This desire to “get high” was a reason why many people abused bupropion, a phenomenon that can be explained by findings from Cooper and colleagues. Cooper et al. compared the activities of bupropion with that of amphetamine to investigate the production of stereotypical activities that characterize amphetamine and found that while the mechanisms differ, bupropion at a high dose induced these stereotypical activities [48]. One of the patients, a nursing student who had learned that the euphoria obtained by abusing cocaine is mediated by dopamine, began to abuse bupropion upon learning that bupropion is a dopamine reuptake inhibitor [30]. Our findings also suggest that patients can go to any length to obtain bupropion to achieve their desired effects. Some of the methods of obtaining bupropion specifically for the purpose of getting high included telling their physician they needed bupropion to help with quitting smoking [20] and insisting on a bupropion prescription to manage depression [28]. These findings suggest that clinicians need to be cautious in prescribing bupropion to patients with a previous or current history of substance use disorder and to warn patients about the potential addictive effect of bupropion.

This case report synthesis showed the variety of adverse effects that can result from bupropion abuse. The most common adverse effect was generalized convulsion while rare fatalities such as death and loss of consciousness were also reported. Other commonly reported adverse effects include headaches, tachycardia, nausea, vomiting, tremor/tremulousness, nasal burning, pain, drooling/rhinorrhea, and bad taste/no effect [22,30,33,38,39]. Other than adverse medical effects, bupropion abuse, especially in a prison setting, is likely to lead to other non-medical issues, although most case reports in this study did not report on them. Because the prison setting is an environment where there are considerable restrictions in the prescription of stimulants and other controlled substances, demand for alternative drugs with stimulant-like effects such as bupropion is inevitable [28]. This demand is an incentive for inmates with medically prescribed bupropion to engage in drug diversion in exchange for money, commodities, or services [11,49]. On the other hand, inmates with prescription bupropion could be victimized, intimidated, and assaulted by inmates seeking to abuse/misuse this drug [49,50]. Such inmate victimization and assault can result in violence that often threatens employees’ physical safety [51]. Drug-seeking behavior or malingering is another possible implication [49]. Although screening procedures for identifying malingering exist [52], inmates skilled in deploying manipulative means could still fall through screening systems [53]. This could cause correctional facilities to implement additional screening measures, thereby increasing the correctional facilities’ work burden [49]. Furthermore, clinicians who deny prescriptions to inmates with abuse potential could be bullied, intimidated, or threatened [54].

The most common route of administration was by intranasal insufflation which was reported in half (50%) of the patients. The other routes used in the cases were oral (32%) and IV (<1%). Because bupropion is not routinely assayed for in toxicology tests, it will be helpful to examine the nostrils of patients for whom there is a high index of suspicion for substance abuse, but who are negative for toxicology screening. Another important finding was that most of the reported bupropion abusers (63.6%) were also people with substance use disorders, suggesting that a history of substance use or multiple substance use is a risk factor for bupropion abuse, and requiring extra care by providers who prescribe bupropion [13,14,19,20,21]. The other common comorbidity found in the cases was mood disorders (54%). However, assessing the relationship between bupropion misuse and mood disorder is difficult owing to the clinical indication of bupropion prescription for depression.

While the number of reported cases of prison-related bupropion abuse at the time of writing this article is still scant, evidence suggests that it is an increasing phenomenon. This increase coupled with the difficulties associated with reporting, researching, and publishing drug abuse in such settings and the presence of observable patterns among bupropion abusers are other reasons why it is imperative to study bupropion abuse in prison populations [22]. Given the high number of case reports on this subject, the variety of settings where they are reported from, and the unique patterns observed in this case report synthesis, there is a high chance that many cases of bupropion abuse are going unreported in different settings. There is a need to investigate substance abuse is already a public health concern in the general population [55]. Like other substances commonly abused by incarcerated individuals, unfettered abuse of bupropion could quickly spiral into societal implications such as increased crime rates, hospitalizations, consumption of public funds, child abuse, and child neglect [56]. These are not without their economic implications, including a high cost of treatment, prevention, morbidity and mortality, labor lost to morbidity and mortality, as well as criminality cost [57].

Limitations

A limitation of this study is the fact that it is an empty systematic review. Many in the research community have the opinion that empty systematic reviews do not bring additional knowledge to what is already known. The authors are, however, of the opinion that this systematic review although did not find any study that meets its criteria is important as it highlights this knowledge gap and the need to address it. A follow-up original study that addresses this gap is being proposed by the authors. Another important limitation of this study is closely related to the first, namely, the sparse reporting of prison-related bupropion abuse. While this article set out to explore studies (and case reports) of bupropion abuse in prison settings, the low number of reported cases of prison-related bupropion abuse presented a significant deficiency, further reinforcing the need to conduct more formal studies on the subject.

## Conclusions

In this systematic review and synthesis of case reports, we demonstrated that various reports of prison and non-prison-related bupropion abuse have been published and described the patterns of abuse in the cases. We also showed that while bupropion abuse is an emerging problem in US prisons as well as in the general population, no original study using primary data has been conducted to estimate the prevalence of this problem in the prison population. These findings point to the need for future research to focus on addressing this area as the lack of research and awareness of this problem may lead to continued abuse of bupropion with its associated adverse medical, social, and economic effects. There is also the need for medical providers that serve prison populations to look out for patients who may feign illness, reject conventional treatments for their depression, or complain of unbearable adverse effects of other medications so as to be prescribed bupropion. Clinicians require a high index of suspicion whenever any patient with a prior history of substance abuse is to be started on bupropion.

## Appendices

**Table 3:** Search strategy for PubMed.

Search	Search string	Search results	2/21/2023
#1	“Substance-Related Disorders”[MeSH] OR “Substance use” OR “substance abuse” OR “substance misuse” OR “drug dependence” OR “drug misuse” OR “drug abuse” OR “drug addict*” OR prescription OR overprescription	452,861	506,194
#2	Bupropion OR wellbutrin	5,109	5,534
#3	“prisoners”[MeSH Terms] OR “prisons”[MeSH Terms] OR prison* OR “correctional facilities”[MeSH Terms] OR “correctional facilities” OR "jails"[MeSH Terms] OR jail* OR penitentiar*	34,150	37,643
#4	#1 AND #2 AND #3	6	6

**Table 4:** Search strategy for Scopus.

Search	Search string	Search results	
#1	ALL ( “Substance-Related Disorders” OR “Substance use” OR “substance abuse” OR “substance misuse” OR “drug dependence” OR “drug misuse” OR “drug abuse” OR “drug addict*” OR prescription OR overprescription )	1,017,332	
#2	( TITLE-ABS-KEY ( bupropion ) ) OR wellbutrin	7,488	
#3	ALL ( prison* OR “correctional facilit*” OR jail* OR penitentiar* )	336,343	
#4	#1 AND #2 AND #3	58	68

**Table 5:** Search strategy for EMBASE.

Search	Search string	Search results	
#1	‘substance-related disorders’ OR ‘substance use’ OR ‘substance abuse’ OR ‘substance misuse’ OR ‘drug dependence’ OR ‘drug misuse’ OR ‘drug abuse’ OR ‘drug addict*’ OR prescription OR overprescription	518,398	1,300,039
#2	Bupropion OR wellbutrin	7,753	21,146
#3	prison* OR ‘correctional facilit*’ OR jail* OR penitentiar*	40,002	46,342
#4	#1 AND #2 AND #3	4	21

**Table 6:** Search strategy fo APA PsycINFO.

Search	Search string	Search results	
S1	TX “Substance-Related Disorders” OR “Substance use” OR “substance abuse” OR “substance misuse” OR “drug dependence” OR “drug misuse” OR “drug abuse” OR “drug addict*” OR prescription OR overprescription	198,042	
S2	TX Bupropion OR wellbutrin	2,409	
S3	TX prison* OR “correctional facilit*” OR jail* OR penitentiar*	34,339	
S4	S1 AND S2 AND S3	5	5

**Table 7:** Search strategy for CINAHL.

Search	Search string	Search results	
S1	TX “Substance-Related Disorders” OR “Substance use” OR “substance abuse” OR “substance misuse” OR “drug dependence” OR “drug misuse” OR “drug abuse” OR “drug addict*” OR prescription OR overprescription	168,097	
S2	TX Bupropion OR wellbutrin	4,086	
S3	TX prison* OR “correctional facilit*” OR jail* OR penitentiar*	31,015	
S4	S1 AND S2 AND S3	21	28

**Table 8:** PRISMA 2020 checklist.

Section and topic	Item #	Checklist item	Location where item is reported
Title			
Title	1	Identify the report as a systematic review.	
Abstract			
Abstract	2	See the PRISMA 2020 for Abstracts checklist.	
Introduction			
Rationale	3	Describe the rationale for the review in the context of existing knowledge.	
Objectives	4	Provide an explicit statement of the objective(s) or question(s) the review addresses.	
Methods			
Eligibility criteria	5	Specify the inclusion and exclusion criteria for the review and how studies were grouped for the syntheses.	
Information sources	6	Specify all databases, registers, websites, organisations, reference lists and other sources searched or consulted to identify studies. Specify the date when each source was last searched or consulted.	
Search strategy	7	Present the full search strategies for all databases, registers and websites, including any filters and limits used.	
Selection process	8	Specify the methods used to decide whether a study met the inclusion criteria of the review, including how many reviewers screened each record and each report retrieved, whether they worked independently, and if applicable, details of automation tools used in the process.	
Data collection process	9	Specify the methods used to collect data from reports, including how many reviewers collected data from each report, whether they worked independently, any processes for obtaining or confirming data from study investigators, and if applicable, details of automation tools used in the process.	
Data items	10a	List and define all outcomes for which data were sought. Specify whether all results that were compatible with each outcome domain in each study were sought (e.g. for all measures, time points, analyses), and if not, the methods used to decide which results to collect.	
	10b	List and define all other variables for which data were sought (e.g. participant and intervention characteristics, funding sources). Describe any assumptions made about any missing or unclear information.	
Study risk of bias assessment	11	Specify the methods used to assess risk of bias in the included studies, including details of the tool(s) used, how many reviewers assessed each study and whether they worked independently, and if applicable, details of automation tools used in the process.	
Effect measures	12	Specify for each outcome the effect measure(s) (e.g. risk ratio, mean difference) used in the synthesis or presentation of results.	
Synthesis methods	13a	Describe the processes used to decide which studies were eligible for each synthesis (e.g. tabulating the study intervention characteristics and comparing against the planned groups for each synthesis (item #5)).	
	13b	Describe any methods required to prepare the data for presentation or synthesis, such as handling of missing summary statistics, or data conversions.	
	13c	Describe any methods used to tabulate or visually display results of individual studies and syntheses.	
	13d	Describe any methods used to synthesize results and provide a rationale for the choice(s). If meta-analysis was performed, describe the model(s), method(s) to identify the presence and extent of statistical heterogeneity, and software package(s) used.	
	13e	Describe any methods used to explore possible causes of heterogeneity among study results (e.g. subgroup analysis, meta-regression).	
	13f	Describe any sensitivity analyses conducted to assess robustness of the synthesized results.	
Reporting bias assessment	14	Describe any methods used to assess risk of bias due to missing results in a synthesis (arising from reporting biases).	
Certainty assessment	15	Describe any methods used to assess certainty (or confidence) in the body of evidence for an outcome.	
Results			
Study selection	16a	Describe the results of the search and selection process, from the number of records identified in the search to the number of studies included in the review, ideally using a flow diagram.	
	16b	Cite studies that might appear to meet the inclusion criteria, but which were excluded, and explain why they were excluded.	
Study characteristics	17	Cite each included study and present its characteristics.	
Risk of bias in studies	18	Present assessments of risk of bias for each included study.	
Results of individual studies	19	For all outcomes, present, for each study: (a) summary statistics for each group (where appropriate) and (b) an effect estimate and its precision (e.g. confidence/credible interval), ideally using structured tables or plots.	
Results of syntheses	20a	For each synthesis, briefly summarise the characteristics and risk of bias among contributing studies.	
	20b	Present results of all statistical syntheses conducted. If meta-analysis was done, present for each the summary estimate and its precision (e.g. confidence/credible interval) and measures of statistical heterogeneity. If comparing groups, describe the direction of the effect.	
	20c	Present results of all investigations of possible causes of heterogeneity among study results.	
	20d	Present results of all sensitivity analyses conducted to assess the robustness of the synthesized results.	
Reporting biases	21	Present assessments of risk of bias due to missing results (arising from reporting biases) for each synthesis assessed.	
Certainty of evidence	22	Present assessments of certainty (or confidence) in the body of evidence for each outcome assessed.	
Discussion			
Discussion	23a	Provide a general interpretation of the results in the context of other evidence.	
	23b	Discuss any limitations of the evidence included in the review.	
	23c	Discuss any limitations of the review processes used.	
	23d	Discuss implications of the results for practice, policy, and future research.	
Other information			
Registration and protocol	24a	Provide registration information for the review, including register name and registration number, or state that the review was not registered.	
	24b	Indicate where the review protocol can be accessed, or state that a protocol was not prepared.	
	24c	Describe and explain any amendments to information provided at registration or in the protocol.	
Support	25	Describe sources of financial or non-financial support for the review, and the role of the funders or sponsors in the review.	
Competing interests	26	Declare any competing interests of review authors.	
Availability of data, code and other materials	27	Report which of the following are publicly available and where they can be found: template data collection forms; data extracted from included studies; data used for all analyses; analytic code; any other materials used in the review.	

## References

[1] Wagner Wagner, Bertram Bertram (2021). “What percent of the U.S. is incarcerated?” (And other ways to measure mass incarceration). https://www.prisonpolicy.org/blog/2020/01/16/percent-incarcerated/#:~:text=The%20answer%3A%20About%200.7.

[2] Glaze L, Kaeble D, Statisticians BJS (2014). Correctional Populations in the United States, 2013. Justice.

[3] (2021). National Institute on Drug Abuse (NIDA). Criminal Justice DrugFacts. National Institute on Drug Abuse.

[4] (2021). Key substance use and mental health indicators in the United States:
results from the 2019 National Survey on Drug Use and Health. Mental Health Indicators in the United States: Results from the.

[5] Degenhardt L, Hall W (2012). Extent of illicit drug use and dependence, and their contribution to the global burden of disease. Lancet.

[6] Fergusson DM, Horwood LJ, Swain-Campbell N (2002). Cannabis use and psychosocial adjustment in adolescence and young adulthood. Addiction.

[7] Angarita GA, Emadi N, Hodges S, Morgan PT (2016). Sleep abnormalities associated with alcohol, cannabis, cocaine, and opiate use: a comprehensive review. Addict Sci Clin Pract.

[8] Brook JS, Richter L, Whiteman M, Cohen P (1999). Consequences of adolescent marijuana use: incompatibility with the assumption of adult roles. Genet Soc Gen Psychol Monogr.

[9] Tucker JS, Ellickson PL, Orlando M, Martino SC, Klein DJ (2005). Substance use trajectories from early adolescence to emerging adulthood: a comparison of smoking, binge drinking, and marijuana use. J Drug Issues.

[10] Bronson J, Stroop J, Zimmer S, Berzofsky M (2017). Drug Use, Dependence, and Abuse Among State Prisoners and Jail Inmates, 2007-2009. BJS: Bureau of Justice Statistics.

[11] Fazel S, Bains P, Doll H (2006). Substance abuse and dependence in prisoners: a systematic review. Addiction.

[12] Paccosi S, Cresci B, Pala L, Rotella CM, Parenti A (2020). Obesity therapy: how and why?. Curr Med Chem.

[13] Stahl SM, Pradko JF, Haight BR, Modell JG, Rockett CB, Learned-Coughlin S (2004). A Review of the neuropharmacology of bupropion, a dual norepinephrine and dopamine reuptake inhibitor. Prim Care Companion J Clin Psychiatry.

[14] Huecker MR, Smiley A, Saadabadi A (2022). Bupropion. https://pubmed.ncbi.nlm.nih.gov/29262173/.

[15] Al-Rousan T, Rubenstein L, Sieleni B, Deol H, Wallace RB (2017). Inside the nation's largest mental health institution: a prevalence study in a state prison system. BMC Public Health.

[16] Stassinos GL, Klein-Schwartz W (2016). Bupropion "abuse" reported to US Poison Centers. J Addict Med.

[17] Doebler S, Ryan A, Shortall S, Maguire A (2017). Informal care-giving and mental ill-health - differential relationships by workload, gender, age and area-remoteness in a UK region. Health Soc Care Community.

[18] Simpson J (2016). Psychopharmacology in jails: an introduction. Carlat Psychiatry Report.

[19] Schifano F, Chiappini S (2018). Is there a potential of misuse for venlafaxine and bupropion?. Front Pharmacol.

[20] Baribeau D, Araki KF (2013). Intravenous bupropion: a previously undocumented method of abuse of a commonly prescribed antidepressant agent. J Addict Med.

[21] Page MJ, McKenzie JE, Bossuyt PM (2021). The PRISMA 2020 statement: an updated guideline for reporting systematic reviews. BMJ.

[22] Naglich AC, Brown ES, Adinoff B (2019). Systematic review of preclinical, clinical, and post-marketing evidence of bupropion misuse potential. Am J Drug Alcohol Abuse.

[23] Farrah K, Young K, Tunis MC, Zhao L (2019). Risk of bias tools in systematic reviews of health interventions: an analysis of PROSPERO-registered protocols. Syst Rev.

[24] Hill S, Sikand H, Lee J (2007). A case report of seizure induced by bupropion nasal insufflation. Prim Care Companion J Clin Psychiatry.

[25] Kim D, Steinhart B (2010). Seizures induced by recreational abuse of bupropion tablets via nasal insufflation. CJEM.

[26] Yoon G, Westermeyer J (2013). Intranasal bupropion abuse: case report. Am J Addict.

[27] Reeves RR, Ladner ME (2013). Additional evidence of the abuse potential of bupropion. J Clin Psychopharmacol.

[28] Hilliard WT, Barloon L, Farley P, Penn JV, Koranek A (2013). Bupropion diversion and misuse in the correctional facility. J Correct Health Care.

[29] Anderson R (2021). Bupropion: the “poor man’s cocaine”? A case report. https://acoep-rso.org/the-fast-track/bupropion-the-poor-mans-cocaine-a-case-report/.

[30] Langguth B, Hajak G, Landgrebe M, Unglaub W (2009). Abuse potential of bupropion nasal insufflation: a case report. J Clin Psychopharmacol.

[31] Welsh CJ, Doyon S (2002). Seizure induced by insufflation of bupropion. N Engl J Med.

[32] Khurshid KA, Decker DH (2004). Bupropion insufflation in a teenager. J Child Adolesc Psychopharmacol.

[33] Rigatti M (2021). Bupropion overdose: suicide attempt or recreational disaster?. https://www.acep.org/toxicology/newsroom/mar2019/bupropion-overdose-suicide-attempt-or-recreational-disaster/.

[34] Al-Saiegh Y, Spears J, De Klerk PS, Hitchings J, Lee C, Mahr T (2021). A rare case of ARDS caused by bupropion inhalation and treated with noninvasive ventilation. Case Rep Crit Care.

[35] Rahman S, Mohiuddin S, LL LL (2013). Seizures secondary to bupropion misuse/abuse: three cases. Adolesc Psychiatry.

[36] Rostas A, Wolf U (2015). Bupropion abuse resulting in hypomania in a geriatric amphetamine user: a case report. Am J Addict.

[37] Vento AE, Schifano F, Gentili F, Pompei F, Corkery JM, Kotzalidis GD, Girardi P (2013). Bupropion perceived as a stimulant by two patients with a previous history of cocaine misuse. Ann Ist Super Sanita.

[38] McCormick J (2002). Recreational bupropion abuse in a teenager. Br J Clin Pharmacol.

[39] Oppek K, Koller G, Zwergal A, Pogarell O (2014). Intravenous administration and abuse of bupropion: a case report and a review of the literature. J Addict Med.

[40] Mercerolle M, Denooz R, Lachâtre G, Charlier C (2008). A fatal case of bupropion (Zyban) overdose. J Anal Toxicol.

[41] Reeves RR, Ladner ME, Perry CL, Burke RS, Laizer JT (2015). Abuse of medications that theoretically are without abuse potential. South Med J.

[42] Marusich JA, Lefever TW, Novak SP, Blough BE, Wiley JL (2013). Prediction and prevention of prescription drug abuse: role of preclinical assessment of substance abuse liability. Methods Rep RTI Press.

[43] Tella SR, Ladenheim B, Cadet JL (1997). Differential regulation of dopamine transporter after chronic self-administration of bupropion and nomifensine. J Pharmacol Exp Ther.

[44] Lamb RJ, Griffiths RR (1990). Self-administration in baboons and the discriminative stimulus effects in rats of bupropion, nomifensine, diclofensine and imipramine. Psychopharmacology (Berl).

[45] Spealman RD, Bergman J, Madras BK (1991). Self-administration of the high-affinity cocaine analog 2β-carbomethoxy-3β-(4-fluorophenyl)tropane. Pharmacol Biochem Behav.

[46] Phillips D (2012). Wellbutrin®: misuse and abuse by incarcerated individuals. J Addict Nurs.

[47] Murray B, Carpenter J, Dunkley C (2020). Single-agent bupropion exposures: clinical characteristics and an atypical cause of serotonin toxicity. J Med Toxicol.

[48] Cooper BR, Hester TJ, Maxwell RA (1980). Behavioral and biochemical effects of the antidepressant bupropion (Wellbutrin): evidence for selective blockade of dopamine uptake in vivo. J Pharmacol Exp Ther.

[49] Burns KA (2009). The top ten reasons to limit prescription of controlled substances in prisons. J Am Acad Psychiatry Law.

[50] Wolff N (2016). A general model of harm in correctional settings. The Oxford Handbook of Prisons and Imprisonment.

[51] Stewart TL, Brown DW (2001). Focusing on correctional staff safety. Corrections Today.

[52] Rogers R (2008). Structured interview of reported symptoms (SIRS). Encyclopedia of Psychology and Law.

[53] McDermott BE, Sokolov G (2009). Malingering in a correctional setting: the use of the Structured Interview of Reported Symptoms in a jail sample. Behav Sci Law.

[54] Ritzman M (2021). Workplace bullying, emotional abuse and harassment in corrections. Special Topics and Particular Occupations, Professions and Sectors.

[55] (2021). Defining and Implementing a Public Health Response to Drug Use and Misuse. Am J Public Health.

[56] Hoffman RS, Goldfrank LR (1990). The impact of drug abuse and addiction on society. Emerg Med Clin North Am.

[57] National Drug Intelligence Center (2011). The Economic Impact of Illicit Drug Use on American Society. Homeland Security Digital Library.

